# Ammonia Gas Sensor Based on Graphene Oxide-Coated Mach-Zehnder Interferometer with Hybrid Fiber Structure

**DOI:** 10.3390/s21113886

**Published:** 2021-06-04

**Authors:** Xiaofeng Fan, Shuying Deng, Zhongchao Wei, Faqiang Wang, Chunhua Tan, Hongyun Meng

**Affiliations:** Guangdong Provincial Key Laboratory of Nanophotonic Functional Materials and Devices, School of Information and Optoelectronic Science and Engineering, South China Normal University, Guangzhou 510006, China; 2017021779@m.scnu.edu.cn (X.F.); 2016021800@m.scnu.edu.cn (S.D.); wzc@scnu.edu.cn (Z.W.); fqwang@scnu.edu.cn (F.W.); tch@scnu.edu.cn (C.T.)

**Keywords:** fiber sensor, graphene oxide, Mach-Zehnder interferometer, gas sensing, ammonia gas

## Abstract

A graphene oxide-coated in-fiber Mach-Zehnder interferometer (MZI) formed with a multimode fiber-thin core fiber-multimode fiber (MMF-TCF-MMF) is proposed and experimentally demonstrated for ammonia gas (NH_3_) sensing. The MZI structure is composed of two segments of MMF of length 2 mm, with a flame-tapered TCF between them as the sensing arm. The MMFs act as mode couplers to split and recombine light owing to the core diameter mismatch with the other fibers. A tapered TCF is formed by the flame melting taper method, resulting in evanescent wave leakage. A layer of graphene oxide (GO) is applied to the tapered region of the TCF to achieve gas adsorption. The sensor operates on the principle of changing the effective refractive index of the cladding mode of a fiber through changing the conductivity of the GO coating by adsorbed NH_3_ molecules, which gives rise to a phase shift and shows as the resonant dip shifts in the transmission spectrum. So the concentration of the ammonia gas can be obtained by measuring the dip shift. A wavelength-shift sensitivity of 4.97 pm/ppm with a linear fit coefficient of 98.9% is achieved for ammonia gas concentrations in the range of 0 to 151 ppm. In addition, we performed a repetitive dynamic response test on the sensor by charging/releasing NH_3_ at concentration of 200 ppm and a relative humidity test in a relative humidity range of 35% to 70%, which demonstrates the reusability and stability of the sensor.

## 1. Introduction

Toxic, flammable and explosive gases are often found in people’s daily lives and production activities, which have major safety accidents. Volatile and toxic ammonia gas is an important component in industrial production [1]. Once a leak occurs, ammonia gas can cause environmental pollution and pose a serious health risk, such as concentrations over 100 ppm rapidly induce irritations of eyes and respiratory tracts and create functional troubles. Therefore, the detection and early warning of ammonia gas is necessary. Since ammonia gas applications are usually in a harsh environment such as corrosive, traditional electrochemical sensors are widely used due to their high measurement accuracy [2,3,4,5], but have many shortcomings such as short life, easy contamination and poor repeatability. A sensor capable of overcoming the above disadvantages and capable of performing timely, effective and stable ammonia gas detection is undoubtedly a research hotspot. Since the advent of fiber-optic sensors, they have attracted much attention. Compared with traditional sensing technologies, fiber-optic sensing technologies have characteristics of simple structure, high sensing sensitivity, small size, anti-electromagnetic interference and distributed online monitoring. As a result, more and more fiber-optic-based ammonia gas sensor have appeared in recent years. According to the principle of sensing, there are sensors based on fiber evanescent fields [6], surface plasmon resonance [7] and Bragg grating [8,9]. Various special types of fibers are also used to make related sensors, such as porous plastic fiber [10], thin core fiber [11], photonic crystal fiber [12] and side-polished fiber [13]. It is also the focus of gas sensors to improve the adsorption and selectivity of the detection gas based on different sensitizing substances. Some of the sensitive materials used for ammonia gas detection are silver nanoparticles/PVP/PVA hybrid [14], Ag/SnO_2_ thin films [15], silver nanoparticles doped silica nanocomposites [16], silica-gel [17], graphene/polyaniline [13], three-dimensional zinc oxide nanoflowers [18] and PANI@SnO_2_ nanocomposite [19]. In recent years, in view of the high sensitivity and response speed of evanescent field caused by microfiber to environmental changes, more and more researchers are paying attention to the microfiber sensor for ammonia gas sensing [20,21,22,23]. However, we have to notice that the difficulty in manufacturing, handling and packaging may limit the application of microfiber sensor. The Mach-Zehnder interferometer (MZI) is widely studied and used to measure many parameters such as humidity [24,25], temperature [26], refractive index [26,27] and gas concentration [11,20,28] due to its characteristics of compact and flexible structure, easy fabrication, high sensitivity, good stability and distributed measurement.

As a new type of two-dimensional nanomaterial, graphene has many unique and excellent properties [29,30,31,32] and has been widely used in the field of sensing [8,13,20,21,28]. Graphene oxide (GO) is an important graphene derivative, which inherits the properties of graphene, such as large specific surface area [29], and has excellent properties such as dispersibility, hydrophilicity and biocompatibility [32,33,34]. It should be noted that GO has a more attractive cost-effective and potential for large-scale production of graphene-based materials compared to graphene [35]. In addition, the surface of GO contains oxygen functional groups such as a hydroxyl group, an epoxy group, a carboxyl group and a carbonyl group [32,36], so that it can adsorbs gas molecules, and thus many studies have been proposed for gas sensing [22,23]. The absorption-related properties of graphene and graphene oxide on ammonia gas molecules have been studied [37,38], in which the results indicate that the GO showed highly distinguished selectivity towards NH_3_ in comparison with H_2_ and CH_4_ gases. The sensing mechanism of graphene materials is mainly due to its two-dimensional structure at the micro-nano level and its large surface area, which is very beneficial to the atomic or molecular level. In full contact, these contacts are essentially charge exchanges between molecules or atoms, which will change the local carrier concentration in the graphene material. Thus, a series of photoelectric properties of graphene such as electrical conductivity, optical frequency, effective dielectric constant and effective refractive index will change [21,39]. If the graphene material is combined with the optical waveguide, the two of them will form a hybrid waveguide, and once the graphene is affected by the external environment, the photoelectric properties of it will change, which will also profoundly affect the propagation field of the optical waveguide [20,40,41].

In this paper, we present a Mach-Zehnder fiber interferometer (MZI) coated with GO for measuring ammonia gas concentration. Based on the Mach-Zehnder interferometer structure, which mainly forms stable and clear interference, we introduce a tapered region to enhance the evanescent field, which is used to improve the perception of the external environment changes in the sensing arm region. The MZI structure is composed of two segments of multimode fibers (MMFs) with a segment of tapered-thin core fiber (TCF) spliced between them. The MMFs act as mode couplers to split and recombine light where the mode fields are mismatched. The tapered-TCF is used as a sensing arm to respond to changes in the ambient conditions. A GO layer is coated onto the surface to improve the sensitivity. The charge exchanges will occur when the GO film absorbs ammonia gas, and thus cause the change of the refractive index of GO. As a result, the effective refractive index of the cladding mode will change. The resonant wavelength of the MZI will shift because the phase difference between the core mode and the cladding mode changes. So, the concentration of the ammonia gas can be obtained by measuring the shift. This paper is organized as follows: the structure and principle of the sensor are introduced in Section 2. The fabrication of the sensing head is presented in Section 3. Section 4 contains the experimental results and discussions. Finally, the paper is ended with some conclusion in Section 5.

## 2. Sensor Structure and Principle

**Figure 1:**
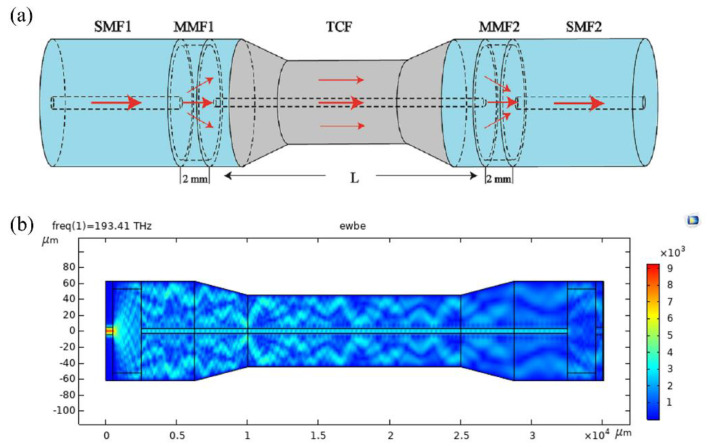
(**a**) Schematic of the proposed MZI with a multimode-fiber-thin core-fiber-multimode-fiber (MMF-TCF-MMF) structure. (**b**) Simulated result of the mode field distribution.

Figure 1a shows the structure of an optical fiber MZI sensor, in which a tapered-TCF is spliced between two MMFs (MMF1 and MMF2) to act as the sensing arm; the MMFs are spliced to the single-mode fiber (SMF1 and SMF2) which play the role as the mode couplers. When light from SMF1 is launched through MMF1, due to the mismatch of the mode field diameter, some is coupled into the cladding of TCF and excites different cladding modes while the remaining light enters the core of the TCF and continues to transmit. In the tapered region of TCF, part of the light in the core and cladding will leak to the external environment in the form of evanescent waves due to the reduction of the fiber diameter. This will cause the tapered area to be very sensitive to the changes of external environment. At the TCF-MMF2 splice point, the cladding modes of the TCF are coupled into the MMF2 core and interfere with each other as well as with the core mode. Subsequently, most of the light are coupled into the core of SMF2. Figure 1b shows the simulated result with the software of COMSOL Multiphysics. It can be found that the beam splitting and coupling occur at the regions of MMFs.

In this paper, we use the MMFs to adjust the number and intensity of excitation modes and the intensity ratio between core mode and cladding modes. However, the MMFs will also cause additional phase differences between the core mode and the cladding modes because the transmission path of them is not exactly same. Thus, the length of the two MMFs should be suitably selected so that the phase difference between their guided modes can be negligible and the cladding modes can be excited sufficiently. The interference is mainly formed by two modes (the core mode and the dominant cladding mode) in the sensor, so the structure of sensor still can be considered as a Mach-Zehnder interferometer. The output intensity and phase difference can be expressed as follows:

(1) $$I=I_{co}+I_{cl}+2\sqrt{I_{co}I_{cl}}cos\varphi$$

where:

(2) $$\varphi =\frac{2\pi \left(n_{co}-n_{cl}\right)}{\lambda }L=\frac{2\pi \Delta n_{eff}}{\lambda }L$$

*I_co_* and *I_cl_* represent the light intensity of the core and cladding mode, respectively, and *φ* is the phase difference of them. The incident wavelength is *λ* and *L* is the length of the tapered-TCF. The effective refractive indexes of the TCF core and cladding mode are *n_co_* and *n_cl_*, respectively, while $\Delta n_{eff}\;$is the difference between them. When the phase difference *φ* = (2*m* + 1) *π* in (1) and m is an integer, the intensity of interference reaches a minimum value and the wavelength of the interference valley in the transmission spectrum is:

(3) $$\lambda_{dip}=\frac{2}{2m+1}\Delta n_{eff}L$$

From (3), one can see that the wavelength of the interference valley $\lambda_{dip}$ will vary with a change in $\Delta n_{eff}$ or *L*. In this paper, a GO film is coated on the surface of the tapered TCF. When the GO absorbs the ammonia gas, the refractive index of it will vary due to the conductivity changing. As a result, the *n_cl_* and $\Delta n_{eff}$ will change and the $\lambda_{dip}$ will shift. So, the concentration of ammonia gas can be measured by the shift of $\lambda_{dip}$.

## 3. Sensing Head Fabrication

### 3.1. Fusion and Tapering of the Sensor

**Figure 2:**
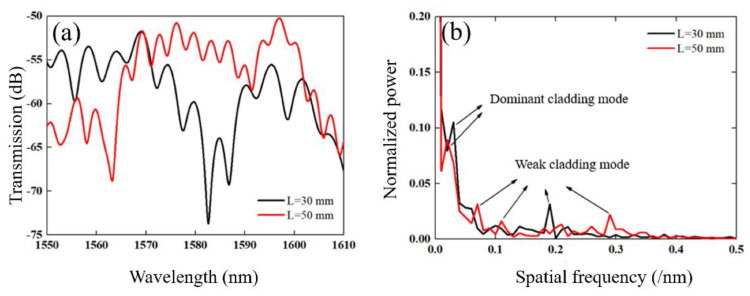
(**a**) Transmission spectrum of the sensor with different TCF lengths (*L* = 30 mm, *L* = 50 mm). (**b**) Spatial frequency of fiber fusion based MZI sensor with different lengths.

As shown in Figure 1, the fibers are spliced in turn with a fusion splicer (FITEL S178, Furukawa Electric, Tokyo, Japan). The arc power and duration time are 140 bit and 3000 ms, respectively. The diameters of the core/cladding of the SMF, MMF and TCF were 9/125 µm, 105/125 µm and 6.4/125 µm, respectively. It should be noted that the MMFs act as mode couplers, and their length affects the excitation and coupling efficiency of the cladding modes. A short MMF cannot excite the cladding mode sufficiently and the coupling efficiency is low. Through repeated attempts and experiments, the length of the two MMFs of 2 mm was selected to obtain a good interference pattern. As the sensing arm, the length of the TCF will affect the free spectral range (FSR) of the interference spectrum. In general, the longer the length of the TCF, the narrower the FSR that can be achieved. Figure 2a shows the transmission spectra for the TCF lengths of 30 and 50 mm, in which the FSR is seen to decrease as the length increases. The inhomogeneous interference fringes also show that several cladding modes have been excited, as shown in Figure 2b. Figure 2b is the Fourier transform from Figure 2a, which can show the spatial frequency characteristics [42,43,44]. We believe that the interference is from the TCF and the spectrum will be affected by the ambient conditions of the TCF. In this paper, the *L* = 30 mm is used for the taper and ammonia gas response experiment.

**Figure 3:**
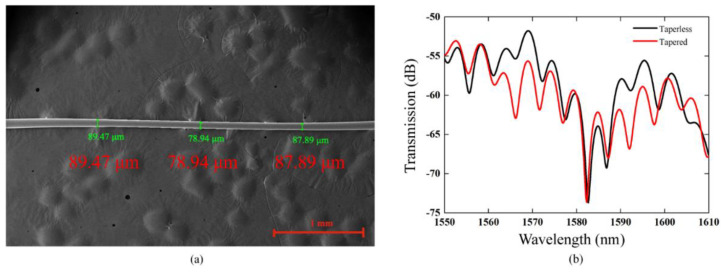
(**a**) SEM image of the tapered-TCF. (**b**) Transmission spectrum of the MZI sensor with and without tapering.

In order to create better coupling into the cladding mode, the TCF is stretched to introduce a taper. The sensing head is fixed on the three-dimensional adjustment frame and the TCF is heated by a torch (PT-220, IRODA, Taiwan) and stretched to obtain a tapered-TCF. Figure 3a shows an SEM image of the TCF after tapering. One can see that the cladding diameter of the tapered TCF has decreased from 125 µm to about 80 µm. The transmission spectrum of the sensor with and without tapering is shown in Figure 3b. After tapered, the contrast ratio increases because the intensity ratio between the core mode and cladding mode varies, meanwhile the dip wavelengths are almost unchanged.

### 3.2. Preparation and Coating of Graphene Oxide

**Figure 4:**
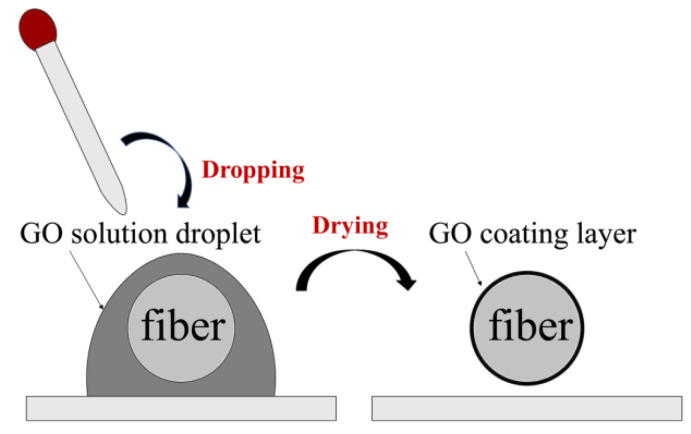
Schematic of coating graphene oxide onto the fiber.

**Figure 5:**
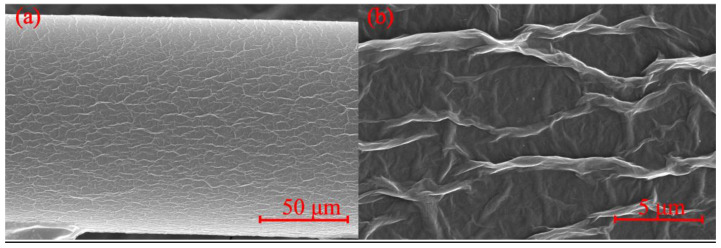
SEM image of the tapered-TCF after coating with GO. Resolution: (**a**) 50 μm, (**b**) 5 μm.

**Figure 6:**
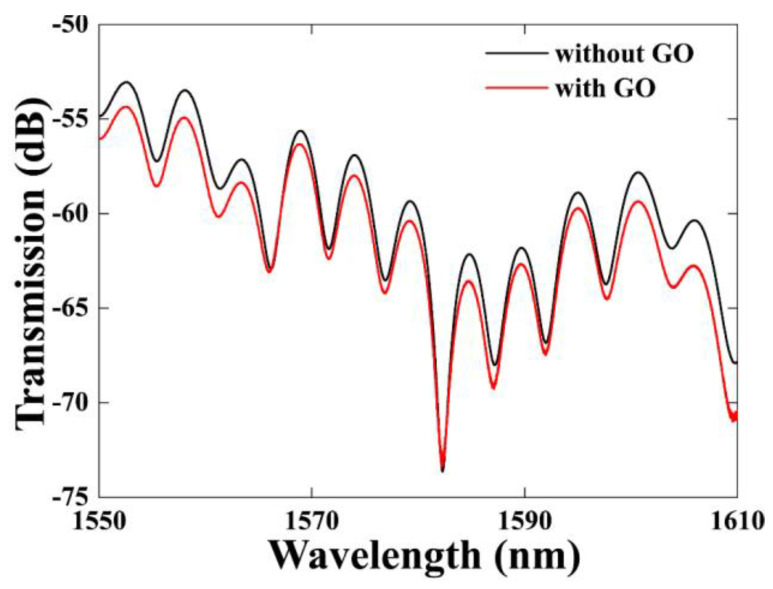
Transmission spectrum before and after coating with graphene oxide.

The process of coating GO onto the tapered-TCF is shown in Figure 4. First, a 0.08 mg/mL GO dispersion (XF020, Nanjing XFNANO Materials Tech. Co., Ltd., Nanjing, China) was centrifuged at 10,000 RPM for 15 min to create GO sheets with a size larger than 500 nm. Second, the fiber was cleaned with alcohol several times, and the sensing area was immersed in a sufficient-sized droplet of GO dispersion and was naturally evaporated for 24 h at temperature of 25 °C. As a result, GO adhered to the surface of the fiber, seen in the SEM image (Figure 5). We observe that the GO layer is uniformly attached to the surface of the tapered-TCF and a large number of undulating folds are formed simultaneously. The undulating folds increase the effective surface area of the GO exposed to the gas, which is advantageous for absorption. It should be noted that the thickness of the GO film can be adjusted by controlling the concentration of the GO solution and the immersion time. For comparison, we measured the transmission spectra of the sensor with and without GO (Figure 6). There are no obvious changes except for the contrast, and the GO coating has not affected the interference properties of the sensor.

**Figure 7:**
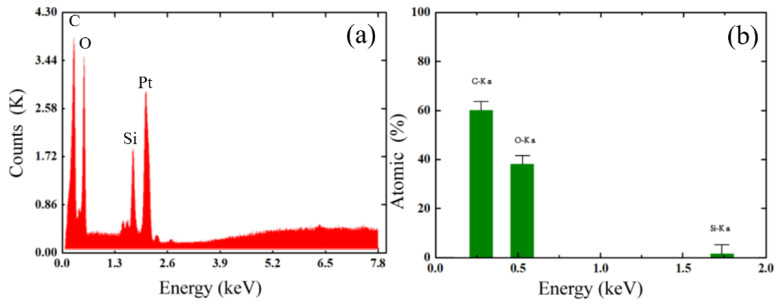
(**a**) EDS of GO coated on the surface of the tapered-TCF. (**b**) Atomic percentage of each major element.

To further determine the elemental composition of the GO coating material on the fiber, we performed an energy dispersive spectroscopy (EDS) analysis, as shown in Figure 7a. The coating material mainly contains the elements C, O, Si and Pt; Si is a component of the optical fiber, and Pt was sprayed on to obtain the EDS image. Figure 7b shows the atomic composition of each major element of the coating material; the specific gravity of the three elements C, O and Si are 59.92, 38.17 and 1.92%, respectively, and the GO coating is without significant impurities. It should be noted that the functional group in the GO contains hydrogen, which does not appear in this EDS elemental analysis.

## 4. Experimental Results and Discussion

**Figure 8:**
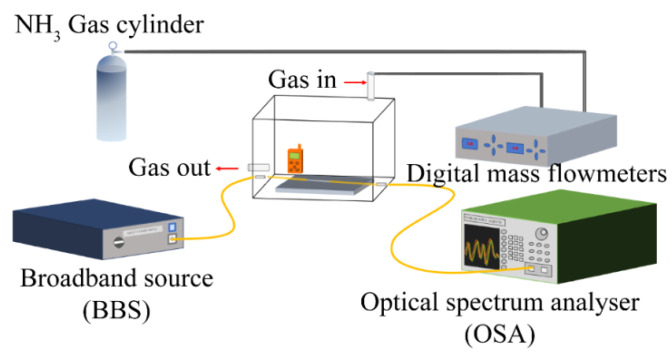
Schematic of gas sensing measurement system.

The performance of this sensor was investigated using the experimental setup shown in Figure 8. The sensor was placed in a sealed gas chamber. A broadband light source (BBS) (ASE-C+L module, HOYATEK, Shenzhen, China) was used to illuminate the interferometer. The output spectrum was acquired by an optical spectrum analyzer (OSA) (AQ6370B, YOKOGAWA, Tokyo, Japan). The gas chamber was constructed of pure silica and kept sealed; the inlet and outlet ports were gas-tight switched valves. Before injecting ammonia gas, the air chamber was connected with the ventilation unit for one hour to discharge as much water vapor and other impurities as possible from the air chamber and the pipeline. The outlet valve was then closed to ensure that the air in the gas chamber was no longer disturbed by external ambient air. At this time, there was only air in the gas chamber, and the output spectrum was recorded as an initial reference for the NH_3_ concentration 0 ppm. Subsequently, the mixture of NH_3_ and N_2_ in a volume ratio of 5:95 was gradually injected into the gas chamber to mix with the air to get different NH_3_ concentrations. The main component of air retained in the air chamber is nitrogen gas, and only nitrogen gas follows a trace amount of ammonia gas into the gas chamber. Further, the molar amount of the injected gas is also small due to the low NH_3_ concentration. So, it can be considered that the change of the transmission spectrum in the experiments is caused by the concentration adjustment of the ammonia gas.

**Figure 9:**
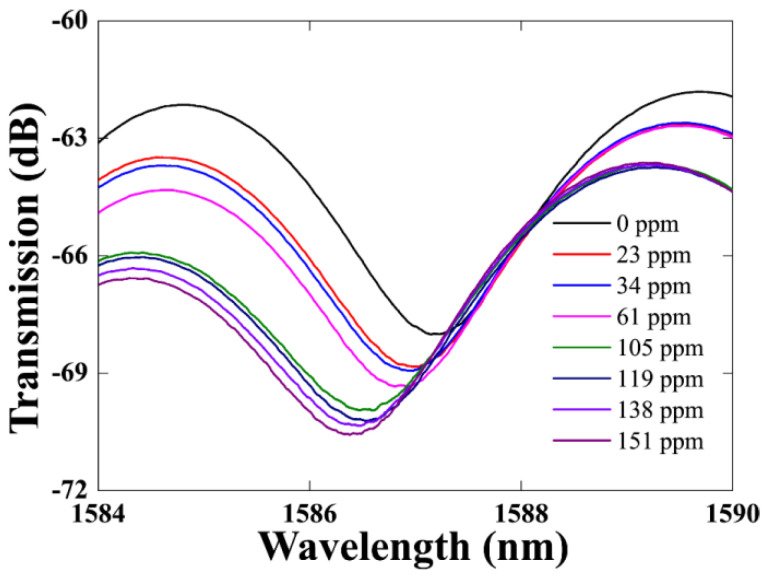
Transmission spectrum of the sensor for ammonia gas concentration from 0 to 151 ppm at temperature of 25 °C.

In the experiments, the ammonia gas concentration increased from 0 to 151 ppm. Figure 9 shows the transmission spectra for different concentrations of NH_3_. The ammonia gas mass flow rate into the chamber was precisely controlled by a digital mass flow meter and the gas concentration was monitored in real time using a chemical ammonia gas sensor placed in the chamber to achieve different ammonia gas concentrations. In order to obtain good observation results, we selected the resonant dip near 1587 nm in Figure 6 as the observation point, and recorded the output spectrum for different ammonia gas concentrations. After the end of the experiment, the gas was discharged from the gas chamber. All experiments were carried out at a temperature of 25 °C and the pressure inside the gas chamber was the same as the atmospheric pressure.

**Figure 10:**
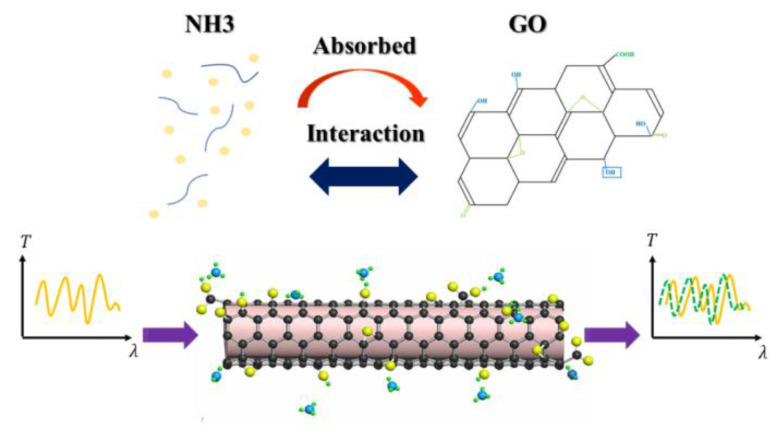
Schematic diagram of NH_3_ sensing mechanism ammonia of the sensor coated GO.

From Figure 9, we can see that the wavelength of the resonant dip shifts to shorter wavelengths as the concentration of NH_3_ increases through the range 0–151 ppm. There are many polar functional groups in GO, such as hydroxyl and carboxyl groups, and epoxy functional groups, which can promote the adsorption of NH_3_ on the GO. GO absorbs NH_3_ and interacts with NH_3_, and the charge transfer from NH_3_ to GO is very significant due to the formation of surface hydrogen bonds [37,39]. The above process will result in a decrease in the dielectric constant and conductivity of GO, so the refractive index of GO will increase because it is inverse proportion to the dielectric constant and conductivity, as shown in Figure 10, and then the effective refractive index of the cladding mode will increase due to the effect by the refractive index change of the GO. As a result, the wavelength of the resonant dip will shift to short wavelength because the effective refractive index difference between the core mode and the cladding mode decreases. In the experiment, the absorption effect will increase when the ammonia gas concentration increases. The phase difference between the core mode and the cladding mode will decrease further, so the wavelength of the resonant dip will continually shift to short wavelength. On the contrary, the wavelength of the resonant dip will shift to near the original position when NH_3_ is desorbed from the GO.

**Figure 11:**
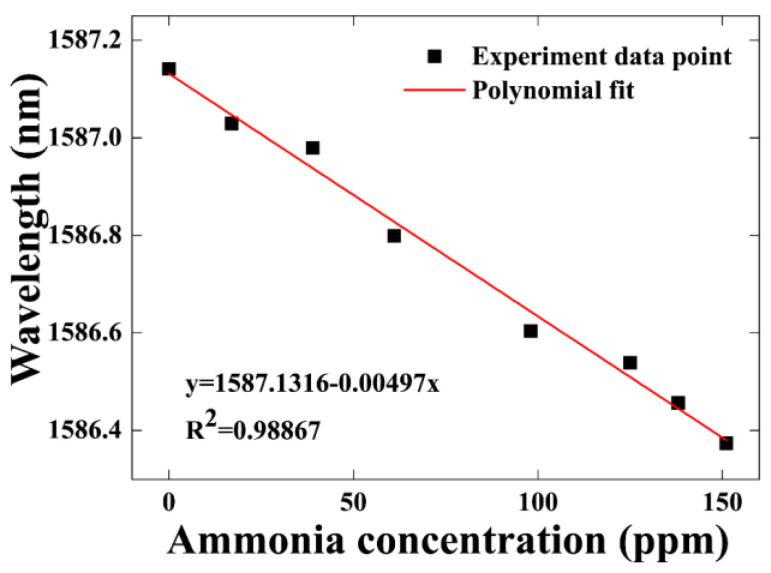
The wavelength shift as a function of ammonia gas concentration from 0 to 151 ppm.

The relationship between wavelength shift and ammonia gas concentration is shown in Figure 11. The linear fitting results indicate a sensitivity of 4.97 pm/ppm with linear fit coefficient of 98.9% can be achieved in the range of 0–151 ppm ammonia gas concentration.

**Figure 12:**
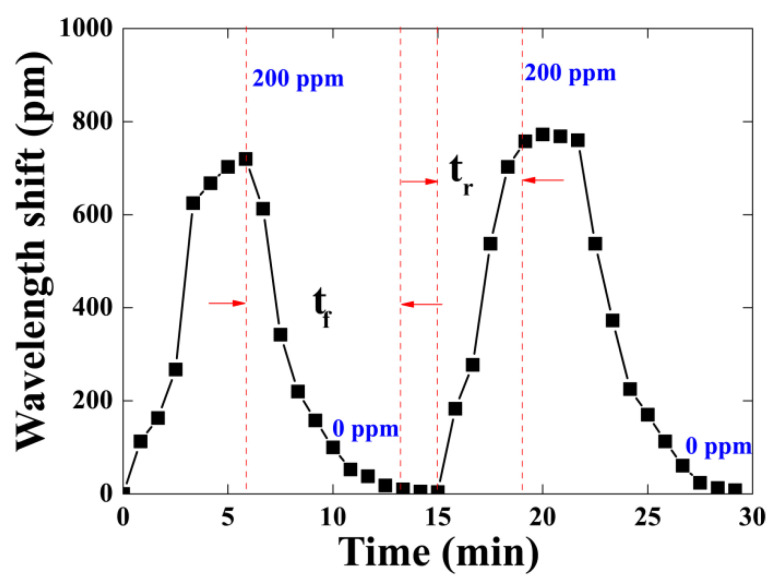
Dynamic responses of the sensor at the ammonia gas concentration of 200 ppm.

In order to investigate the reproducibility and reversibility of the proposed sensor, we repeatedly injected and extracted the ammonia gas with a concentration of 200 ppm into the gas chamber at a temperature of 25 °C. The transmission spectrum was recorded at intervals of 50 s to determine the dynamic behavior of the gas sensor. After the transmission spectrum reached a stable state, indicated when the wavelength of the interferometer minimum was no longer shifting, the next fill or discharge of ammonia gas took place. We recorded the wavelength shift of the resonant dip during two periods of ammonia gas fill and discharge, as shown in Figure 12. The results indicate that the dynamic response rising time (t_r_) and falling time (t_f_) of the sensor are about 5 and 7.5 min for the two periods, respectively. These repeated cycles of exposure to 0 and 200 ppm of NH_3_ gas demonstrate that the rising and falling time are not very fast, but the sensor exhibits good reversibility. The response and recovery time of the sensor are determined by the thickness of the GO layer, the diameter of the tapered fiber and the rate of ammonia gas mixing in the chamber on injection or expulsion.

**Figure 13:**
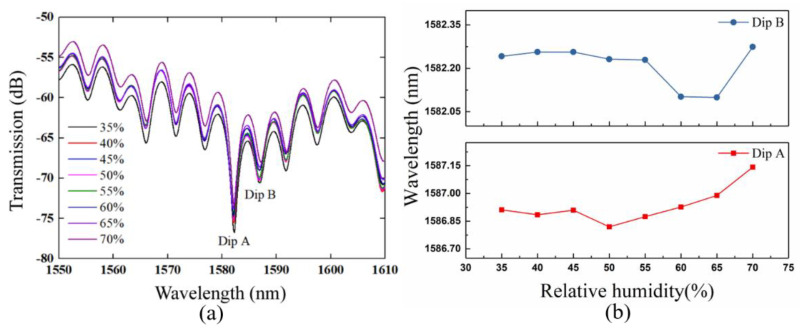
(**a**) Transmission spectrum of the sensor at the relative humidity from 35% to 70%. (**b**) Wavelength fluctuation of dip A and B at the relative humidity from 35% to 70%.

Humidity is a common environmental variable which can also cause transmission spectrum changes, so we have tested the humidity response characteristics of the sensor. The sensor was put into a constant temperature and humidity chamber (J-TOPH-22-B, Jiexin Testing Equipment Co., Ltd., Dongguan, China) with a relative humidity range from 35% to 70%, which basically covers the range of common relative humidity changes. The output transmission spectrum at different relative humidity with the ammonia gas concentration of 0 ppm is shown in Figure 13a. We can see that the wavelength and intensity of the resonant dip will vary when the relative humidity increases from 35% to 70%. GO films are highly hygroscopic due to the presence of oxygen-containing groups on the surface and edges of GO [18,34]. When the relative humidity of the environment increases, the GO film absorbs more water molecules. The water molecules will adhere to the surface of the GO or fill the GO layer, which causes the GO film to swell [25]. Thus, the refractive index of the GO film will decrease as the water molecules increase, and the refractive index difference between the GO film and the fiber cladding will be smaller. When light in the fiber passes through the cladding and reaches the interface between the cladding and the GO film, part of the light is reflected at the interface, and evanescent wave leakage is suppressed and reduced. As a result, the contrast ratio of the transmission spectrum decreases as the relative humidity increases.

We select the Dip A and B to observe the wavelength variation, shown as in Figure 13b. The wavelength fluctuation of 0.23 and 0.14 nm for Dip A and B is not obvious. Furthermore, the relative humidity in the gas response experiments varies hardly. So, we think that the response of the sensor for ammonia gas is obvious.

The performances of the fiber sensors for ammonia gas measurement is listed in Table 1. It can be seen that that the proposed sensor has a high sensitivity in a wide range of RH.

## 5. Conclusions

This paper proposes a GO-coated Mach-Zehnder fiber interferometer for measurement of ammonia gas concentration. When the GO film absorbs ammonia gas, the change in the refractive index of the film affects the effective refractive index of the cladding mode and the phase of light in the fiber. As a result, the wavelength of the dip in the interferometer response is shifted. The results show that a sensitivity of 4.97 pm/ppm with the linear fit coefficient (R^2^) of 98.9% can be achieved in the ammonia gas concentration range of 0–151 ppm. The rising time and falling time of the sensor are about 5 and 7.5 min, respectively. The sensitivity can be improved with a thick GO film, but the response time will also increase simultaneously. It has good linear response and stable room temperature recovery. The proposed sensor has the advantages of simple manufacture, small volume, passive design and can be used for on-line monitoring of ammonia gas in a room temperature atmosphere.

## Figures and Tables

**Table 1:** Performances of optical fiber humidity sensors proposed in different literatures.

Fiber Structure	Material	Sensitivity	Response Time	Recovery Time	Range	Reference
microfiber hybrid waveguide	graphene	~6 pm/ppm	~0.5 s	none	40–360 ppm	[20]
microfiber Bragg grating	graphene	4 pm/ppm	~10 min	~15 min	0–100 ppm	[21]
MZI with a long-period fiber gratings	graphene	~3 pm/ppm	270 s	none	10–180 ppm	[28]
MZI	zinc oxide nanoflowers	5.75 pm/(μg/L)	~50 s	~80 s	0–5500 μg/L	[18]
MZI based PCF	PANI@SnO_2_	none	7 s	2 s	0–8 ppb	[19]
MZI	GO	4.97 pm/ppm	5 min	7.5 min	0–151 ppm	This paper

## Data Availability

The data that supports the findings of this study are available from the authors on reasonable request, see author contributions for specific data sets.
